# A Poly (Caprolactone)-Cellulose Nanocomposite Hydrogel for Transdermal Delivery of Hydrocortisone in Treating Psoriasis Vulgaris

**DOI:** 10.3390/polym14132633

**Published:** 2022-06-28

**Authors:** Pierre P. D. Kondiah, Thankhoe A. Rants’o, Sipho Mdanda, Lauwrence M. Mohlomi, Yahya E. Choonara

**Affiliations:** Wits Advanced Drug Delivery Platform Research Unit, Department of Pharmacy and Pharmacology, School of Therapeutic Sciences, Faculty of Health Sciences, University of the Witwatersrand, Johannesburg, 7 York Road, Parktown 2193, South Africa; pierre.kondiah@wits.ac.za (P.P.D.K.); thankhoe.rantso@wits.ac.za (T.A.R.); sipho.mdandah7@gmail.com (S.M.); matsosolauwrence@gmail.com (L.M.M.)

**Keywords:** hydrogels, nanoparticles, drug delivery, psoriasis vulgaris, polymers

## Abstract

Psoriasis vulgaris (PV) is a common chronic disease, affecting much of the population. Hydrocortisone (HCT) is currently utilized as a PV treatment; however, it is associated with undesirable side effects. The aim of this research was to create a thermo-responsive nano-hydrogel delivery system. HCT-loaded sorbitan monostearate (SMS)-polycaprolactone (PCL) nanoparticles, encapsulated with thermo-responsive hydrogel carboxymethyl cellulose (CMC), were synthesized by applying the interfacial polymer-deposition method following solvent displacement. The nanoparticles’ properties were evaluated employing Differential Scanning Colorimetry, Thermogravimetric Analysis, Fourier Transform Infrared Spectroscopy, Scanning Electron Microscopy, Zeta sizer, Ultraviolet/Visual spectroscopy, and cytotoxicity testing. The nanoparticle sizes were 110.5 nm, with polydispersity index of 0.15 and zeta potential of −58.7 mV. A drug-entrapment efficacy of 76% was attained by the HCT-loaded SMS-PCL nanoparticles and in vitro drug-release profiles showed continuous drug release over a period of 24 hrs. Keratinocyte skin cells were treated with HCT-loaded SMS-PCL nanoparticles encapsulated with CMC; the results indicated that the synthesized drug-delivery system was less toxic to the keratinocyte cells compared to HCT. The combined trials and results from the formulation of HCT-loaded SMS-PCL nanoparticles encapsulated with CMC showed evidence that this hydrogel can be utilized as a potentially invaluable formulation for transdermal drug delivery of HCT, with improved efficacy and patient conformity.

## 1. Introduction

Chronic inflammatory skin diseases are mediated by complex immunological mechanisms. In psoriasis, the hyperproliferation of keratinocytes triggers an immune response, leading to the release of cytokines [1,2]. Presently, there is no known cause for this disease, as it can be triggered by a plethora of factors. It is considered an immune-mediated disorder with immune cells, such as T-cells and dendritic cells, as well as a pro-inflammatory agent, tumor necrosis factor-α, playing a major role in its pathogenesis [3]. Psoriasis affects up to approximately 5% of the world population, and has been declared as a serious non-communicable disease by the World Health Organization [4]. Psoriasis vulgaris (PV) is the most prevalent form of this disease, accounting for 90% of all psoriasis diagnoses [4]. The clinical presentations of PV include scaly itching, oedema, fatigue, burning, and erythema. In addition to the physical manifestations, this disease has also been linked with a decline in mental state, leading to suicidal thoughts and an overall deterioration in quality of life [5,6]. Current treatment interventions for PV include topical corticosteroids, phototherapy, and systemic therapies, based on the severity of the disease. Topical therapy forms the backbone of PV treatment for mild to severe cases. South African treatment guidelines state corticosteroids as a standard of care for this disease, and they are also on the list of essential medicine, maintained by the World Health Organization (WHO) [7]. Hydrocortisone (HCT) is a class V–VII, low-to-intermediate-potency corticosteroid with anti-pruritic, anti-inflammatory, and vasoconstrictive effects. While the anti-pruritic and anti-inflammatory effects directly suppress the epidermal mitosis and inflammation associated with PV, the vasoconstriction within the upper dermis indirectly reduces inflammation by decreasing the blood flow to the inflammation site, hence, the quantity of inflammatory mediators that are delivered to this area of the skin is affected. The immunosuppressive activity of HCT includes inhibition of the humoral factors implicated in conferring an inflammatory response, such as the immunoglobulin G antibody, suppression of T-cell proliferation, and dendritic cell maturation as well as the prevention of leukocyte migration to the cutaneous inflammation sites [3,8]. As a result, HCT is indicated for use (topically) in the treatment of PV [8]. At a molecular level, HCT interacts with steroid receptors in the skin, mainly the glucocorticoid and mineralocorticoid receptors [8,9]. Generally, steroid receptors have three domains, including a DNA-binding domain, N-terminal transactivation domain and a ligand-binding domain, where steroid hormones bind [9,10]. Upon HCT binding, the ligand-receptor complex translocates from the cytoplasm to the nucleus, where they cause transcription and expression of various genes [11]. Both of these receptors are important in the pathogenesis and treatment of PV. They collectively work to regulate the development and curbing of the skin inflammation in PV [11]. It has been shown that the glucocorticoid deficiency causes skin inflammation and proliferation in PV. This state is effectively reversed by topical treatment with steroids such as HCT [12].

Currently, HCT is prescribed in the form of ointments, creams, and lotions for relief of PV symptoms. HCT strength in topical creams normally ranges between 0.1–2.5% [13]. These HCT formulations show efficacy as therapeutic agents against PV; however, they are associated with undesirable local and systemic side effects, such as skin atrophy, pruritus, erythema, skin dryness, and suppression of the Hypothalamus–Pituitary–Adrenal gland (HPA) axis [13]. Other factors contributing to the limited utilization of topical corticosteroids are: a lack of compliance by patients, frequent application of the medication, cost and availability of the medication, and a lack of adequate knowledge about the disease [14]. In this study, HCT was employed as model anti-psoriatic drug for the treatment of PV. To overcome challenges of patient compliance as well as to safeguard against the undesirable side effects of HCT, novel and efficacious drug-delivery systems should be investigated. Novel transdermal drug carriers have been evaluated for enhancing the skin penetration of corticosteroids. Examples of this include microemulsion hydrogels, niosomes, liposomal hydrogels, deformable liposomes, and solid-lipid nanoparticles [15,16]. This study aimed to synthesize HCT-loaded sorbitan monostearate (SMS)-polycaprolactone (PCL) lipid nanoparticles encapsulated with carboxymethyl cellulose (CMC) hydrogel, to form a hydrogel that is responsive to temperature (body temperature ~37 °C) and pH (skin pH ~5.2), for improved transdermal treatment of PV.

Hydrogels are popular in biological applications because of their low invasiveness and capacity to deliver medicines [17]. Due to their low cytotoxicity and mild inflammation in vivo, hydrogels have critical implications for reconstructive surgery, tissue engineering, and drug-delivery applications [17]. Ziai et al. (2022), mentioned that bimolecular sensing is one of the most exciting areas in the field of smart biopolymers. As a result, multifunctional biomimetic, biocompatible, and stimuli-responsive hydrogel materials have piqued the curiosity of researchers [18]. Hydrogels are hydrophilic-polymer networks that can absorb 10–20% (an arbitrary lower limit) of their dry weight in water. Hydrogels can be chemically stable, or they can deteriorate, disintegrate, and dissolve. When the networks are linked together by molecular entanglements and/or secondary forces, such as ionic, H-bonding, or hydrophobic interactions, they are referred to as ‘reversible’ or ‘physical’ gels [19]. Gels are semi-solid formulations of organic polymers obtained through the dispersion of liquid molecules within a solid medium, resulting in supramolecular, three-dimensional (3D) assemblies. Molecules of the solid component generally undergo strong intermolecular bonding (physical force) or form chemical bonds that result in the cross-linking of the solid molecules to form a 3D architecture [20,21]. Hydrogels as a method of drug delivery are gaining a considerably high interest [22]. In contrast to conventional therapy, hydrogels as topical drug-delivery systems have several advantages, including improved patient’s quality of life, sustained drug release, and drug absorption by surmounting the skin barrier [23]. Thermo-responsive hydrogels are hydrogels that respond to temperature as a stimulus [24]. Thermo-responsive hydrogels synthesized from natural polymers are more advantageous, as they are biocompatible and biodegradable as compared to their synthetic counterparts [25].

Sorbitan monostearate (SMS) is a hydrophobic, non-ionic surfactant used as an emulsifier and forms an organogel of organic solvents (e.g., hexadecane, isopropyl myristate, and a range of vegetable oils, such as sesame oil, groundnut oil, olive oil, soybean oil, and sunflower oil) [26,27]. This compound has been evaluated as a potential drug-delivery vehicle. In a study conducted by Singh et al. (2015), the development of SMS and sesame oil-based organogels for topical drug-delivery antibacterial substances found that an SMS–sesame oil combination can be considered as a probable matrix for a controlled release of antimicrobials for topical application [20].

Polycaprolactone (PCL) is a biodegradable polymer composed of short-chain fatty acid repeat units; due to its chemical and mechanical properties, PCL has been extensively investigated in conjunction with a large range of other polymers for its applicability in advanced drug delivery [28]. PCL is hydrophobic in nature, with high solubility (at room temperature), and, owing to its low melting point of ~60 °C, is easily processable. For this reason, PCL presents an attractive potential to researchers to study potential applications in the biomedical field [28]. PCL is a linear polyester with good biocompatibility and a relatively slow degradation rate. In this study, the hydrophobicity of PCL is modified by introducing highly hydrophilic biocompatible material as CMC. Previous studies have already proved its biocompatibility and water affinity are two characteristics that make this compound a potentially functional additive to be introduced in a PCL matrix, to increase its hydrophilicity for biomedical applications [29]. Wang and colleagues studied the double-network Poloxamer 407 (P407) and CMC hydrogel combination for the treatment of atopic dermatitis (an inflammatory skin condition) [30]. In their study, they found that the P407 hydrogel reinforced with CMC yielded positive results. This hydrogel was able to release the model drug at temperatures near to the physiological temperature into localized site of the diseases [30]. The inflamed area of the psoriasis, after administration of the gel, serves as the peripheral stimulus regulating the activation of the response to release the therapeutic agent; hence, in the present study, thermo-responsive polymer CMC will be utilized [31]. The hydrogel formulations of corticosteroids, such as mometasone furoate, have been shown to be an effective method of drug delivery due to high efficacy and a low adverse-response profile [32].

Cellulose is the most abundant polymer occurring in nature and is found as the main component of plant-cell walls [33]. Naturally occurring cellulose is hydrophobic; however, cellulose derivatives are hydrophilic, allowing for their use as hydrogel components [33]. Cellulose derivatives have been extensively investigated for their use as vehicles in drug-delivery research due to their biocompatibility and biodegradability, and their “smart” polymer behaviour, with the ability to respond to temperature as a stimulus [34]. Wang et al. (2016) demonstrated the effective utilization of carboxymethyl cellulose (CMC), an extensively studied cellulose derivative, in combination with P407 as a possible drug-delivery platform in atopic dermatitis [30]. CMC can also be used as a co-polymer in hydrogels to aid in the design of a suitable hydrogel structure. In a study conducted by Nita et al. (2020), co-polymerization of poly (2-Dimethylaminoethyl methacrylate) with CMC confirmed its use as a potential drug-delivery system for transdermal patches, by lowering the LCST from 42–47 °C to 37 °C [35]. CMC are heat-insensitive, under physiological temperatures [36]. Furthermore, CMC biopolymer is an anionic pH-sensitive water-soluble polysaccharide, with numerous single-bond COOH and single-bond OH-functional groups in its structure, which is extensively employed in the fabrication of hydrogel-based drug carriers [37]. Further investigations of this polymer need to be carried out for its potential use in psoriasis vulgaris.

## 2. Materials and Methods

### 2.1. Materials and Equipment

The following chemicals were sourced and utilized throughout this study: acetone, ethanol, methanol, Hydrocortisone (HCT), sorbitan monostearate (SMS), polysorbate 80 (Tween 80), distilled water (dH_2_O), hydrochloric acid (HCl), stearic acid, oleic acid, sodium hydroxide (NaOH), Carboxymethyl Cellulose (CMC), Trypan blue dye, polycaprolactone (PCL), phosphate buffered saline (PBS), Dulbecco’s Modified Eagle’s Medium (DMEM), Trypsin, Dimethyl sulfoxide (DMSO), dimethylimidazole diphenyl tetrazolium bromide (MTT), and mannitol. All chemicals utilized were sourced from Sigma Aldrich (St. Louis, MO, USA) and were of high analytical and experimental grade.

The following equipment was sourced and utilized throughout this study: nanophotometer (Implen^™^ NanoPhotometer^™^ NP80 UV/Vis Spectrophotometer (Munich, Germany)), DSC (Mettler Toledo, DSC1, STARe Instrument (Schwerzenback, Switzerland)], FTIR [PerkinElmer Spectrum 100, Beaconsfield, UK) TGA [PerkinElmer TGA 4000, Beaconsfield, UK], Zeta sizer [Zeta-sizer Nano-ZS machine (Malvern Instruments, Worcestershire, UK)), sonicator (Scientech Ultrasonic Cleaner, Labotec, South Africa], lyophilizer (FreeZone^®^ 2.5, Labconco^®^, Kansas City, MS, USA), incubator (RS Biotechnological Galaxy, Irvine, UK), and inverted light microscope, Model CKX53 (Olympus, Tokyo, Japan).

### 2.2. Spectrophotometric Determination of λmax of HCT and Calibration Curve

Determination of the linear range (calibration curve) was done using standard solutions of the HCT drug at varying concentrations. The sample concentrations ranged from 1–10 µg/mL. The samples were loaded into cuvettes and analyzed utilizing a Implen^™^ NanoPhotometer^™^ NP80 UV/Vis Spectrophotometer (Munich, Germany). The concentration values used were 1 µg/mL, 3 µg/mL, 5 µg/mL, 8 µg/mL, and 10 µg/mL. Each sample was analyzed in triplicate at λmax. To blank the spectrophotometer, 50% ethanol solution was used. The averages (*n* = 3) of the resulting absorbance readings were recorded in the calibration curve [33].

### 2.3. Synthesis of the SMC-PCL Nanoparticle Carrier System

**Figure 1:**
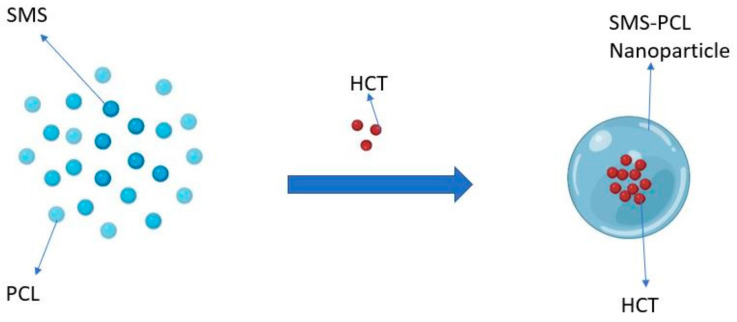
Illustration of the microemulsion method. The figure demonstrates loading of HCT into a micellar structure (SMS-PCL nanoparticle) for loading into the hydrogel (image was completely re-drawn, inspired by Güngör et al. [41]).

Synthesis of the SMS-PCL nanoparticles was performed using a microemulsion technique wherein the hydrophobic corticosteroid HCT was encapsulated within a micelle structure (lipid core) as illustrated in Figure 1. Microemulsions are a system of oil, water, and surfactant. This technique yields isotropic, thermodynamically-stable, and translucent drug-nanoparticle dispersions [38]. This was performed as described by Bender et al. and Da Silva et al., respectively [39,40]. The organic phase was created by accurately weighing 100 mg PCL, 20 mg HCT, and 38 mg SMS dissolved in 25 mL acetone under magnetic stirring at 37 °C until fully dissolved. Separately, an aqueous phase was created by slowly adding Tween 80 up to a final volume of 3 mL into 50 mL water under continuous magnetic stirring until homogenized. Both solutions were combined and left for 10 min under magnetic stirring at room temperature. The same procedure was followed to create the control sample for this study (SMS-PCL blank). The acetone and excess water were removed by rotary evaporation and air drying to concentrate the nanoparticle samples. A final volume of 25 mL of both the HCT-loaded SMS-PCL nanoparticles and blank SMS-PCL nanoparticle samples, respectively, was obtained. The obtained solutions were divided evenly into petri dishes and stored at −80 °C for 24 h. To preserve the samples, a 5% (*w*/*v*) solution of mannitol was created and added in a 1:1 ratio with the nanoparticle samples. Finally, the samples were lyophilized employing a lyophilizer for 24 h at −80 °C to remove any excess water and yield a powder, which was subsequently stored at room temperature throughout the experiment.

### 2.4. Physical Assessment of the SMS-PCL Nanoparticles in Terms of Size, Stability, and Charge Distribution

The size, stability, and polydispersity index (PdI) the nanoparticles were determined using a Zeta sizer Nano-ZS machine (Malvern Instruments, Worcestershire, UK). Briefly, 5 mg of each lyophilized sample was dissolved in distilled water and filtered through 0.22 µm syringe filter (Millipore Corp., Bedford, MA, USA). The samples were transferred to cuvettes and sonicated to ensure complete dissolution. The analyses were performed in triplicates. All readings were performed at 25 °C. Results were obtained using dynamic light scattering and recorded [42,43].

### 2.5. Determination of the Drug-Entrapment Efficacy of HCT within the SMS PCL Nanoparticles

The drug-entrapment efficacy (DEE) of HCT within the SMS-PCL nanoparticles was determined using an indirect method. In short, the SMS-PCL-nanoparticle dispersions (both drug-loaded and blank) were placed in centrifuge tubes and centrifuged at 12,000 rpm for 45 min. The supernatant was carefully withdrawn without disturbing the pallet and diluted utilizing a 50% ethanol solution (at a 1:9 [*v*/*v*] ratio). The samples were analyzed from the calibration curve utilizing a Implen^™^ NanoPhotometer^™^ NP80 UV/Vis Spectrophotometer (Munich, Germany) at wavelength 245 nm making use of the SMS-PCL blank, with loaded supernatant as the blank. To determine the DEE of HCT within the nanoparticles, Equation (1) was used [44,45]:

(1) $$\mathrm{DEE}\;\left(\%\right)=\frac{{\left[\mathrm{Drug}\right]}_{\mathrm{after}\;\mathrm{loading}}}{{\left[\mathrm{Drug}\right]}_{\mathrm{before}\;\mathrm{loading}}\;}\times 100$$

### 2.6. Chemical Evaluation Undertaken on the SMS-PCL Nanoparticles, HCT, and HCT-Loaded Nanoparticles

FTIR spectroscopy was carried out on the HCT, HCT-loaded nanoparticle, and nondrug-loaded nanoparticles (SMS-PCL blank) to compare and evaluate structural properties. FTIR spectra were recorded on a Perkin Elmer Spectrum 100 FTIR spectrometer (Llantrisant, Wales, UK), using an ATR-FTIR cell and a diamond-crystal internal-reflection element. Samples were analyzed at a wavenumber range of 650–4000 cm^−1^ with a resolution of 4 cm^−1^ and 64 scans per spectrum. The resulting spectra were recorded.

### 2.7. Thermal Transition Evaluation of the SMS-PCL Nanoparticles, HCT, and HCT-Loaded Nanoparticles

Comparative DSC analyses were performed on lyophilized samples of the HCT-loaded nanoparticles, HCT drug, and the blank nanoparticle sample using a Mettler Toledo, DSC1, STARe System (Schwerzenback, Switzerland) at a heating rate of 10 °C/min from 0 °C to 200 °C under a constant flow of N2 gas. Accurately weighed samples (10–15 mg) were placed into a covered aluminium sample holder containing a central pin hole. Indium metal (99.99%) was used to calibrate the DSC modulus in relation to temperature and enthalpy. An empty sample holder was used as a reference. DSC thermograms were then compared for transitions.

### 2.8. Thermal Evaluation of the SMS-PCL Nanoparticles, HCT, HCT-Loaded Nanoparticles

TGA was utilized to facilitate rapid degradation analysis by approximating the speed at which these samples become degraded and the specific heat at which this takes place. TGA was carried using the TGA software (PerkinElmer STA 6000, Beaconsfield, UK) to elucidate the thermal characteristics of HCT, HCT-loaded nanoparticles, and blank nanoparticle samples. Ten mg of each sample was weighed accurately and placed on crucibles in preparation for analysis. Heating was undertaken from 30 °C to 900 °C at a rate of 10 °C/min under constant N_2_ gas flow of 20 mL/min.

### 2.9. Synthesis and Evaluation of the CMC Hydrogel System

The method of synthesis of the hydrogel was adopted from Andonova et al. [46]. Briefly, CMC hydrogel was prepared by dissolving 2 g of CMC in 100 mL of boiling deionized water under slow magnetic stirring until the CMC was fully dissolved. Dropwise addition of NaOH was made until gelation occurred.

The temperature it takes for the hydrogel to reach sol-gel transition (SGTT) was determined using a tube inversion method. Briefly, 5 mL of the hydrogel formulation was placed in glass vials and heated in a water bath slowly (rate of 1 °C/min) from 10 °C to 40 °C. The flowability of the hydrogel was observed at each time point by tilting the vial. The temperature at which the liquid is found to be immobile was recorded as the SGTT.

### 2.10. In Vitro Drug Release Undertaken on the SMS-PCL Nanoparticles as Well as the Loaded CMC Hydrogel

The in vitro release of HCT from the HCT-loaded SMS-PCL nanoparticles, as well as the hydrogel system, was investigated using a dialysis method by weighing 40 mg of the HCT-loaded nanoparticles into a dialysis membrane (MW = 10,000 kDa) pre-swollen in 50 mL of PBS (dissolution medium) at pH ~5.2 and pH ~5.9-to simulate the PV infected skin and healthy skin environments, respectively [47,48]. The nanoparticles were also added to 10 mg of the hydrogel to investigate the drug release of HCT from the thermo-responsive CMC hydrogel. Once loaded, the mixture was placed in an isothermal shaker (20 rpm) at 37 °C. Time intervals for sampling were determined and sampling was done over a period of 24 h. At each time point, 3 mL aliquots of the release medium were withdrawn and replaced with an equal volume of fresh medium. The concentration of HCT in the release medium was assayed by UV/Vis spectrophotometry (Implen^™^ NanoPhotometer^™^ NP80 UV/Vis Spectrophotometer, Munich, Germany) at wavelength 245 nm [43,45]. All sampling was performed in triplicate for statistical significance. Calculations of the mass of drug released were performed utilizing the calibration curve and plotted. To determine the mass of the drug released, and percentage thereof, Equation (2) was used:

(2) $$\mathrm{Mass}\;\mathrm{of}\;\mathrm{drug}\;\mathrm{released}\;\left(\mathrm{mg}\right)=\frac{\mathrm{Concentration}\;\mathrm{of}\;\mathrm{drug}\times \mathrm{volume}\;\mathrm{of}\;\mathrm{dissolution}\;\mathrm{medium}}{1000}$$

### 2.11. In Silico Analysis of the HCT Interaction with Glucocorticoid and Mineralocorticoid Receptors

Molecular modelling is an effective computational method used to assess binding profile of a ligand to a receptor [49]. For reliable binding affinity prediction and accurate ligand poses, the molecular mechanics-based approach using implicit solvation strategy, such as molecular mechanics-generalized born surface area (MM-GBSA) re-docking, is employed. Unlike general docking that assumes a stable receptor and flexible ligand, this method is more realistic in providing for flexibility in both the ligand and a receptor while simulating a ligand binding in a continuous solvation model [49,50].

The study to assess molecular interactions of HCT and its steroid receptors was conducted with Schrödinger’s Glide 2021-3 software, Maestro version 12.9, (Schrödinger LLC, New York, NY, USA). The human glucocorticoid and mineralocorticoid receptors were retrieved from the Protein Data Bank (PDB IDs: 4P6X and 5HCV, respectively) [51]. These were individually prepared by the protein-preparation wizard tool. The preparation included removing water molecules beyond 5 Å from the binding site, while leaving those water molecules with possibility to influence ligand binding. Non-hetero groups were also removed, and eventually the protein was optimized at pH 7.0 using PROPKA and minimized by OPLS3e force field to fix all molecular overlaps and strains [52]. The receptor grids were generated based on the previously reported binding sites [53]. The ligand, HCT, was retrieved from PubChem and prepared using the LigPrep (ligand preparation) function. The ligand was subjected to a vigorous pKa-prediction tool, Epik, to generate possible tautomeric and ionization states at pH 7.0 (±2.0) before minimization with OPLS3e force field [52,54].

The initial docking of HCT to the receptors was conducted with Glide’s robust extra precision docking mode to assess the binding interactions and favorable ligand conformations [52]. Following this, the rigorous MM-GBSA re-docking simulation was used to assess and rank the binding affinity of HCT at glucocorticoid and mineralocorticoid receptors. The previously generated extra precision complexes were retrieved and subjected to MM-GBSA under the solvation model VSGB 2.0 together with OPLS3e force field. Finally, the protein distance from ligand was changed from 0 Å to 5 Å to ensure sufficient receptor flexibility during the simulation [52,55].

### 2.12. Analysis of the Surface Morphology of the HCT-Loaded Nanoparticles Using SEM

The shape and structure of the HCT-loaded nanoparticles was studied using Scanning Electron Microscopy (SEM)—JEOL 840 SEM (JEOL, Tokyo, Japan). The lyophilized samples were fixed to a metal stub using conductive tape and then sputter coated with gold. This occurred under vacuum at 2 kV accelerating voltage. The resulting micrographs were evaluated for the surface morphology.

### 2.13. In Vitro Cytotoxicity

#### 2.13.1. Keratinocytes Culture Employing HaCaT Cell Lines

Cytotoxicity evaluation was conducted using the HaCaT cell lines. HaCaT cells are a spontaneous, immortalized human keratinocyte model widely used in the study of skin diseases, including chronic inflammatory skin conditions [56]. The cells were cultured in Dulbecco’s Modified Eagle’s Medium (DMEM) growth medium, supplemented with 10% Foetal Bovine Serum (FBS) and 1% Penicillin-Streptomycin, to form a differentiated model of the human keratinocytes. The HaCaT culture was incubated at 37 °C and 5% CO_2_ saturation under humid conditions (RS Biotechnological Galaxy, Irvine, UK) for 24 h until confluence of >90% was reached, at which stage the cells were sub-cultured. The sub-culturing process was begun by removing growth medium and rinsing the cells with PBS. The cells were detached from the bottom of the T75 cell culture flask using Trypsin. The cells were left to trypsinize for 5 min at 37 °C and 5% CO_2_ and transferred to flasks containing fresh growth medium.

#### 2.13.2. Cell Morphology and Cell Count

The cells were observed for morphology and number using an inverted microscope. Subsequently, the cells were prepared for counting by diluting 0.5 mL of cells in 1 mL DMEM. From the resulting cell suspension, 10 µL was withdrawn and strained with trypan blue dye (1:1 *v*/*v* ratio). Counting was performed utilizing a haemocytometer (1), which was used to count the average number of viable cells.

(3) $$\mathrm{No}.\;\mathrm{of}\;\mathrm{viable}\;\mathrm{cells}\;=\;\mathrm{Avg}\;\mathrm{cells}\;\mathrm{counted}\times \mathrm{Dilution}\;\mathrm{Factor}\times 10^{4}\;\mathrm{cells}/\mathrm{mL}$$

where 10^4^ is a constant with dilution factor being 6.

#### 2.13.3. Standard Drug Preparation

Standard drugs/samples were prepared by dissolving pure HCT, HCT-loaded nanoparticles, and blank nanoparticles in dimethyl sulfoxide (DMSO), to reach a final concentration of 20 mg/mL and 10 mg/mL for 5-fluorouracil (5-FU), respectively (stock solutions). An antineoplastic chemotherapeutic drug, 5-FU, is used as a positive control in this present study. All drugs were vortexed until dissolving completely and stored. The drug-loaded and blank hydrogels were prepared by loading 0.5 mL of the hydrogel (at room temp) with 0.5 mL of the HCT-loaded nanoparticles and 0.5 mL of the blank nanoparticles, respectively, ensuring the two mix thoroughly.

All samples were serially diluted in PBS to achieve concentrations of 1625 µg/mL, 3.25 µg/mL, and 6.5 µg/mL. Different concentration ranges were evaluated in order to establish a relationship between the toxicity of the drug and the cellular response.

#### 2.13.4. In Vitro Cytotoxicity Assay Preparations

The HaCaT cells were seeded in a 96-well plate at a seeding density of 100,000 cells/mL. To each well, 90 µL of the cell suspension was added and incubated at 37 °C, 5% CO_2_ in a humidified atmosphere for 24 h to ensure attachment of the cells to the plate. Samples were treated with 10 µL volumes, making final concentrations of 1.625, 3.25, and 6.5 µg/mL. Following incubation, the cells were treated with 10 µL of the pure HCT, HCT-loaded nanoparticles, HCT-loaded hydrogel, and blank hydrogel. Negative control samples included blank nanoparticles, DMSO (at 0.03%), and untreated cells. For a positive control, cells were treated with 10 µg/mL of 5-Fluorouracil (5-FU). The treated cells were incubated for a further 24 h. All treatments were performed in triplicates and reported as mean values.

#### 2.13.5. MTT Cytotoxicity Assay to Determine the Safety and Tolerability of the HCT-Loaded CMC Hydrogel

Methylthiazole tetrazolium salt (MTT) is a colorimetric assay utilized to determine cytotoxicity and assess cell viability [57]. The in vitro cytotoxicity of the thermos-responsive HCT-loaded hydrogel system was evaluated by employing this assay after 24 and 48 h treatments. Briefly, following the 24-hour incubation period of the treated cells, the growth medium was removed and replenished with fresh medium (90 µL) and addition of 10 µL of the MTT solution (5 mg/mL) to each well. The 96-well plate was incubated for 4 h following addition of MTT at 37 °C and 5% CO_2_ under humid conditions. The resulting formazan crystals that formed at the bottom of the plate were dissolved with 100 µL of the solubilizing agent and incubated overnight at 37 °C and 5% CO_2_. The following day, absorbances were read at 570 nm with a reference wavelength of 620 nm using a multimode microplate reader (Victor X3, Perkin Elmer, MA, USA). Mean values of the corrected absorbance measurements of each triplicate treatment were computed relative to the mean values of DMSO (0.03%) control (set at 100% viability).

The cell viability (%) for each drug concentration was calculated the following equation:

(4) $$\mathrm{Cell}\;\mathrm{viability}\;\left(\%\right)=\frac{\mathrm{Absorbance}\;\left(\mathrm{Test}\right)}{\mathrm{Absorbance}\;\left(\mathrm{Control}\right)}\times 100$$

where Absorbance is the mean absorbance (*n* = 3) recorded for different drug concentrations, and DMSO (0.03%) is taken and used as the control.

#### 2.13.6. Light-Microscopy Analysis to Evaluate Internalization of the Therapeutic Compounds within the HaCaT Cells

The internalization of the HCT-loaded SMS-PCL nanoparticles, HCT-loaded CMC hydrogel, plain hydrogel, SMS-PCL nanoparticles (blank), pure HCT, PBS, DMSO, and 5-FU into the HaCaT cells was determined microscopically. Briefly, after 48 h of treatment, the images were captured using an inverted light microscope, Model CKX53 (Olympus, Tokyo, Japan), using the 10× objective. The resulting images were recorded and presented in the Results section.

## 3. Results and Discussion

### 3.1. Spectrophotometric Analysis Undertaken to Determine λ_max_ of HCT and Calibration Curve

**Figure 2:**
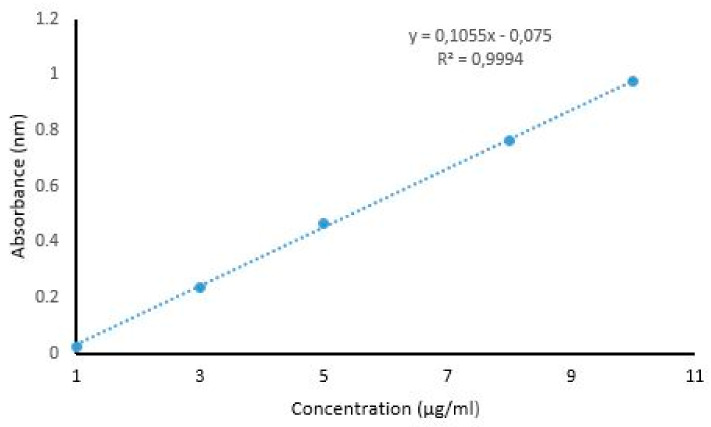
Calibration curve of HCT, undertaken for in vitro evaluation of the formulated samples.

The λmax of HCT was found at 245.0 nm. Based on the λmax, the calibration curve was obtained (Figure 2). This curve confirms that there is a linear correlation between the concentration of the drug and the absorbance (R^2^ = 0.9994)—i.e., an increase in the concentration of the drug results in an increase in the respective absorbance [58].

### 3.2. Physical Assessment of the SMS-PCL Nanoparticles in Terms of Size, Stability, Charge Distribution, and Drug Entrapment

**Figure 3:**
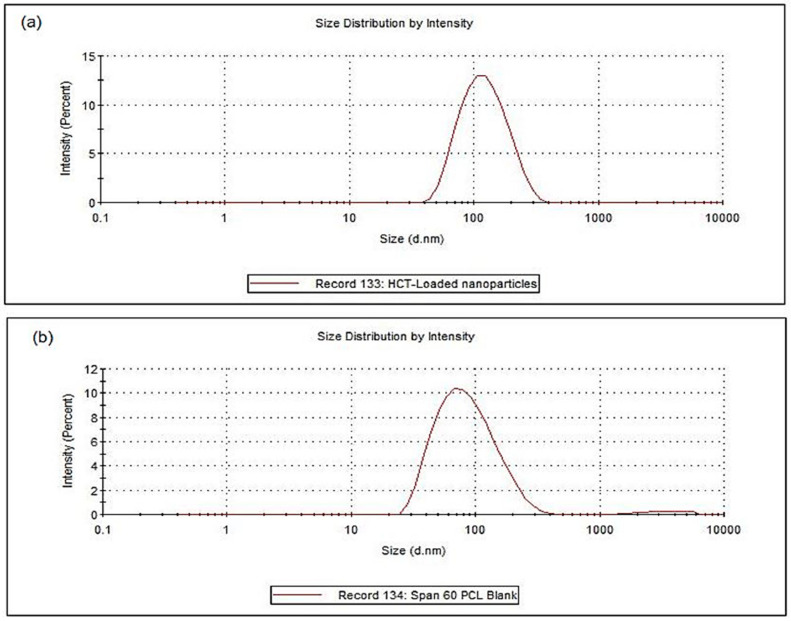
Nanoparticle size of (**a**) HCT-loaded and (**b**) SMS-PCL blank nanoparticles.

The HCT-loaded as well as the blank mean nanoparticle sizes were found to be 110.5 nm and 77.9 nm with PdI of 0.15 and 0.22, respectively. The loading of HCT into the nanoparticles resulted in a larger diameter of the NLC. The mean zeta potential for the HCT-loaded nanoparticles obtained was −58.7 mV as indicated in Figure 3.

Polymeric nanoparticles present the ability to encapsulate and shield compounds against degradation in unfavourable conditions. Lipid nanoparticles can encapsulate hydrophobic drug substances such as the corticosteroids within hydrogels. The size of the nanoparticles is critical to the biological function of these nanoparticles. In the case of drug delivery across the skin (transdermal drug delivery), the optimum particle size for drug delivery to the deep layers of the skin is noted to be <200 nm in diameter [59]. The HCT-loaded nanoparticles were found to be 110.5 nm in diameter, thus making them effective transdermal drug-delivery platforms. The zeta potential of nanostructures is a key indicator of the stability of colloidal-nanostructure dispersions. The mean zeta potential of the HCT-loaded nanoparticles (−58.7 mV) indicates that the nanoparticles have excellent long-term stability. The PdI values recorded for both the HCT-loaded and blank nanoparticles (PdI < 0.5) show that the distribution of the nanoparticle size is of a homogenous nature [43].

The DEE of the HCT-loaded nanoparticles was found to be 76%. Thus, the microemulsion technique proved to be an effective technique for loading HCT in lipid carriers.

### 3.3. Chemical Evaluation Undertaken on the SMS-PCL Nanoparticles, HCT, and HCT-Loaded Nanoparticles

**Figure 4:**
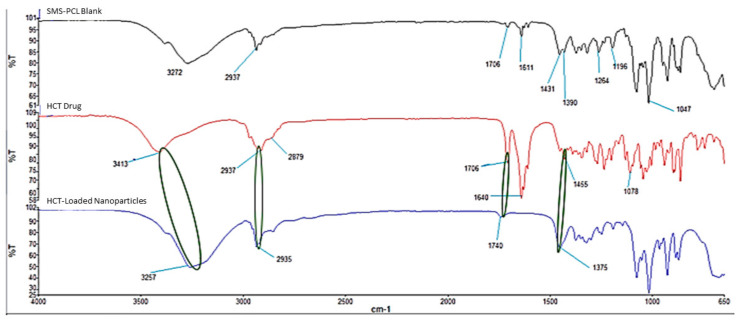
FTIR Spectra of the SMS-PCL blank polymers, HCT drug (pure), and HCT-loaded nanoparticles.

FTIR spectra for HCT, HCT-loaded nanoparticles, and blank (SMS-PCL) samples were obtained and recorded in Figure 4.

The IR spectrum of HCT showed distinct peaks at 3413 cm^−1^, which confirms the presence of –OH groups; aliphatic C–H stretching was observed at 2937 cm^−1^ as well as 2879 cm^−1^, C=O was observed at 1706 cm^−1^ and 1640 cm^−1^, –CH2 bending was seen at 1455 cm^−1^, and, lastly, the spectrum showed C–O at 1078 cm^−1^ [42,60]. In the spectrum for the SMS-PCL blank, the peak observed at 2937 cm^−1^ is due to CH_2_ vibrations, the peak at 1706 cm^−1^ is due to C=O vibrations, and the peak at 1611 cm^−1^ represents O–H vibrations, while the vibrations caused by CH_2_ can be observed at the 1431 and 1390 cm^−1^ peaks, C–O and C–C stretching vibrations can be observed at 1264 cm^−1^ and 1196 cm^−1^, respectively, and COO vibrations are shown at 1047 cm^−1^ [61,62].

With regards to the FTIR spectrum of the drug-loaded nanoparticles, changes in the concavity and intensity of the peaks denotes interaction of the drug with the polymers. In this instance, this interaction was observed at peaks 3257 cm^−1^, where the O–H group of the HCT interacts with the polymer matrix [63]. At 2935 cm^−1^, the spectrum denotes long-chain linear aliphatic compounds’ (SMS) interaction with HCT, followed by more interactions at 1740 cm^−1^ and 1375 cm^−1^, respectively.

### 3.4. Thermal-Transition Evaluation of the SMS-PCL Nanoparticles, HCT, and HCT-Loaded Nanoparticles

**Figure 5:**
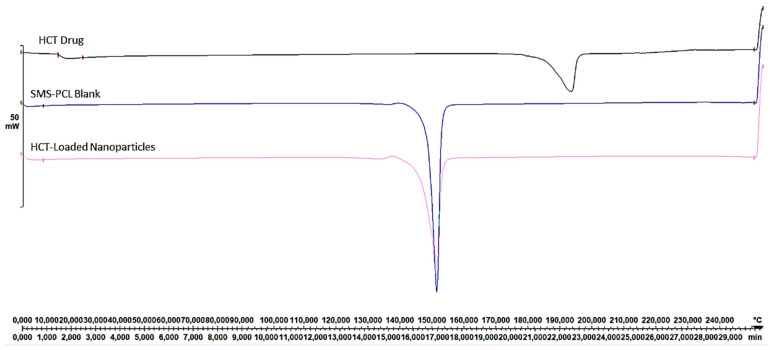
DSC of HCT, SMS-PCL blank, and HCT-loaded nanoparticles.

Comparative DSC analyses were performed on all samples. The thermograms obtained were recorded in Figure 5. In the figure, an endothermic peak can be observed at 200 °C, this corresponds with the melting point of HCT at 22 min; this can be corroborated as within the normal melting range of this drug (200–220 °C), per the literature [14,54,55] and confirmed by TGA data. The HCT melting peak is not observed within the polymer, suggesting that this compound is in an amorphous state, further supporting the fact that it has integration within the SMS-PCL nanoparticulate structure [64]. The SMS-PCL nanoparticles demonstrated a sharp peak at 150 °C after 17 min. No secondary peaks were observed, suggesting that the structure melted completely at this temperature [65,66].

### 3.5. Thermal Evaluation of the SMS-PCL Nanoparticles, HCT, and HCT-Loaded Nanoparticles

**Figure 6:**
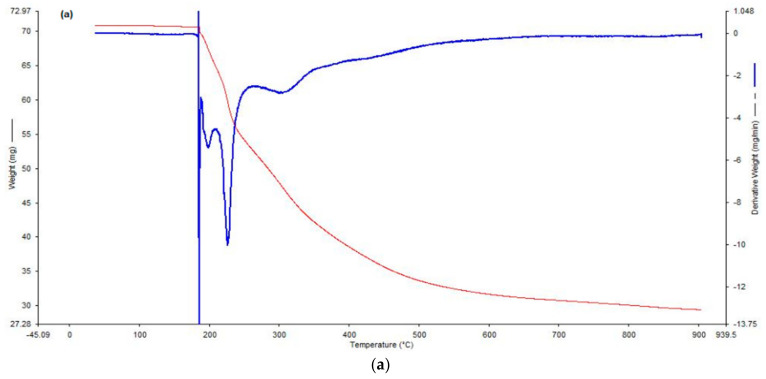

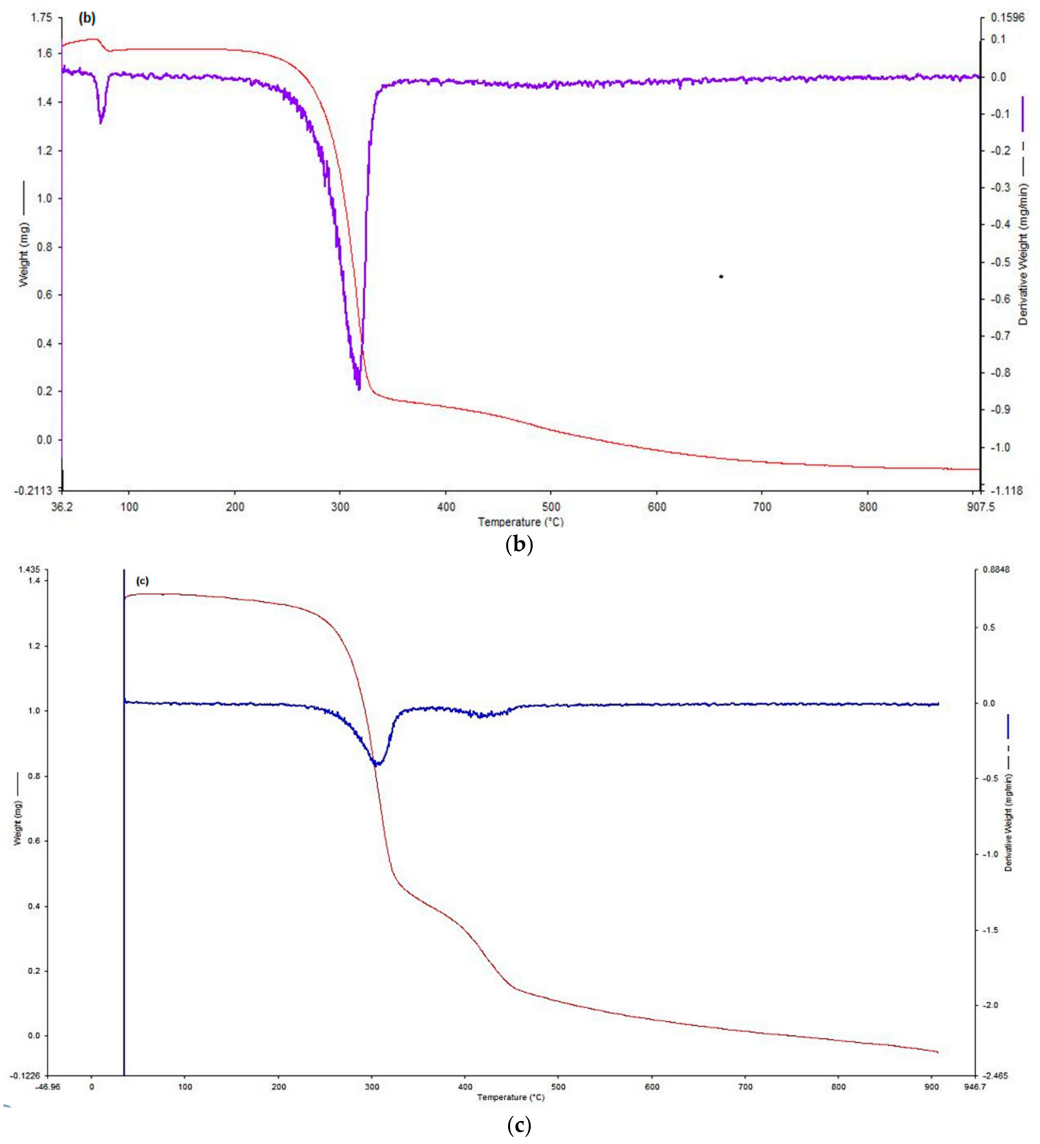
TGA thermograms of (**a**) HCT, (**b**) SMS-PCL blank, and (**c**) HCT-loaded nanoparticles.

TGA thermograms of pure HCT drug, SMS-PCL blank nanoparticles, and HCT-loaded nanoparticles were undertaken. As seen in Figure 6, the derivative weight (mg/min) peaks represent the critical temperatures in which loss of mass and decomposition occur. With regards to the pure HCT drug thermogram (Figure 6a), three critical weight-loss stages can be observed at 220 °C (onset of decomposition), 305 °C (major decomposition), and, lastly, at 630 °C, where the maximum degradation of HCT is seen. In Figure 6b, the SMS-PCL blank samples show mass loss around 60 °C, which could be as a result of the evaporation of organic solvents and water weight. The polymer shows onset of degradation at approximately 290 °C and maximum decomposition between 320 °C and 500 °C. The HCT-loaded nanoparticles (Figure 6c) exhibit similar thermal behaviour to the blank nanoparticles. In the HCT-loaded thermogram, the degradation of HCT drug was not observed [67].

### 3.6. Evaluation of the CMC Hydrogel System for Sol-Gel Transition Temperature

The CMC hydrogel was successfully synthesized. A clear, translucent fluid was obtained. The SGTT was determined using a tube-inversion method. With increasing temperature, the viscosity of the gel increased. The SGTT was determined at ~33 °C ± 2 °C. At room temperature, the hydrogel reverts to liquid consistency. This confirms the successful synthesis of a polymeric thermo-responsive-hydrogel system, capable of the delivery of drugs at physiological temperatures.

### 3.7. In Vitro Drug Release Undertaken on the SMS-PCL Nanoparticles as Well as the Loaded CMC Hydrogel

**Figure 7:**
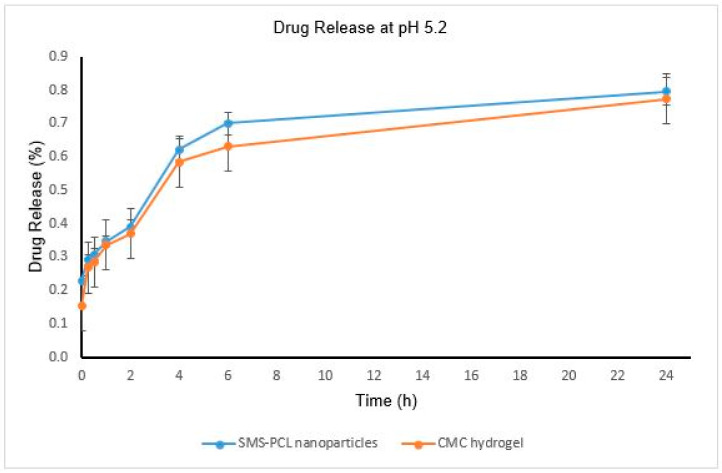
HCT release profiles from SMS-PCL nanoparticles and CMC hydrogel, at simulated PV skin pH (pH 5.2) at 37 °C over a period of 24 h.

**Figure 8:**
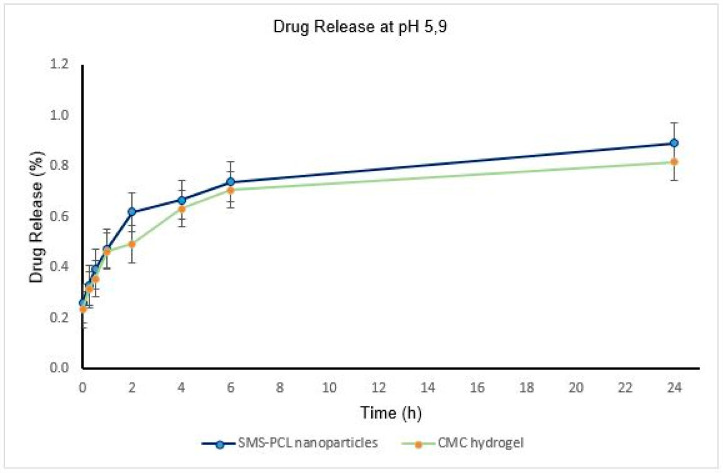
HCT release profiles from SMS-PCL nanoparticles and CMC hydrogel, at simulated normal skin pH (pH 5.9) at 37 °C over a period of 24 h.

The drug-release profiles of HCT from nanoparticles, as well as nanoparticle-loaded hydrogel, were determined by analysis of the release medium at pre-determined time points (Table 1 and Table 2 and Figure 7 and Figure 8) over a period of 24 h. Overall, the HCT drug release from the thermo-responsive CMC hydrogel was slower, compared to the drug release from the nanoparticles in both pH environments. This could be as a result of slow diffusion of the drug across the polymer matrix; this is further evidence of the sustained drug release of HCT across the CMC hydrogel [44]. The maximum concentration of release was observed to be 3.25 µg/mL (0.84% release), which could be attributed to the PCL polymer’s ability to hold onto hydrophobic compounds, such as HCT. The SMS-PCL nanoparticles encapsulated with CMC hydrogel demonstrated a slow, sustained, and controlled release of HCT [68,69]. This is critical in reducing the potential for dermal toxicity and the amount that is available for systemic absorption. The rate of transdermal permeation is proportional to the drug’s concentration in the vehicle. Similarly, dermal toxicity of HCT is dependent on its percutaneous absorption [70]. Moreover, approximately 2% of HCT is known to be absorbed into the systemic circulation from epidermal application [70,71]. The sustained HCT release will also prevent frequent applications of the drug, further reducing the toxicity potential [70,71]. The CMC hydrogel achieves minimal absorption of HCT when applied via the topical route [68]. This is key in preventing the adverse reaction experienced with a high concentration of HCT [72]. This is further exemplified by the currently available formulations of HCT (cream, ointments) having low drug strength in terms of percentage (usually 0.1–2.5%) [6]. The high affinity of steroid receptors to HCT permits the generation of diverse cellular responses from minute concentrations of the drug [9]. A study recently published on skin pH and psoriasis found that psoriatic patients had a lower pH (5.2 ± 0.5). However, no differences in skin pH were observed across the body sites examined (forehead, malar region, chin, volar arm, plaque/elbow) [73,74]. Skin pH is primarily determined by the area of skin and age, but it is also influenced by sex, race, and the time of day when values are measured, and its average value is between 5.4–5.9 [75]. In this study, pH 5.2 and pH 5.9 were evaluated.

It has also been observed that the HCT drug releases at a faster rate at higher pH (5.9)—which is closer to physiological pH—compared to the low pH for both the nanoparticles and hydrogel formulations [69,76]. The results are presented as mean ± SD (*n* = 3).

### 3.8. In Silico Analysis of the HCT Interaction with Glucocorticoid and Mineralocorticoid Receptors

**Figure 9:**
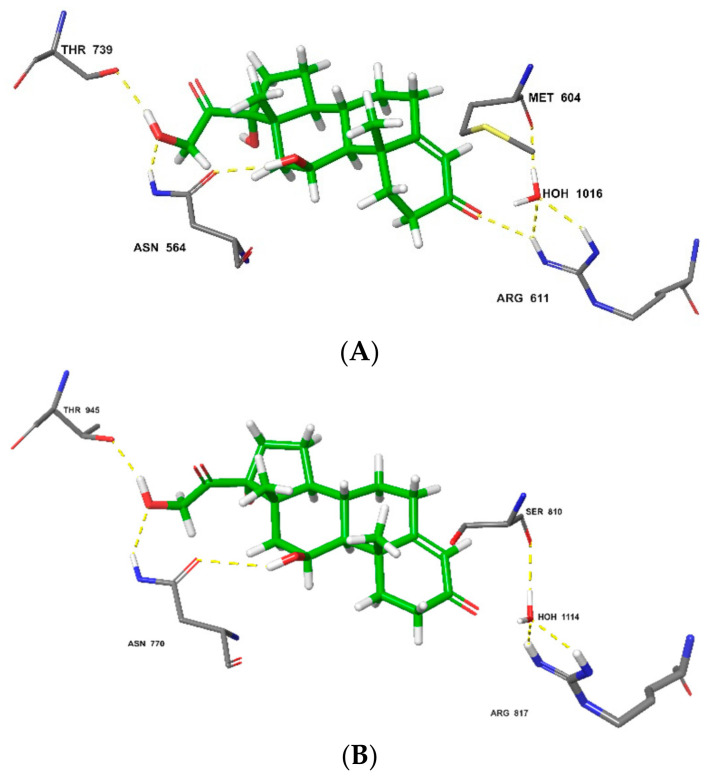
Molecular interactions of HCT and glucocorticoid receptor with (**A**) glide-docking score: −13.026 kcal/mol; and mineralocorticoid receptor with (**B**) glide docking score: −11.084 kcal/mol.

Molecular docking of HCT on the glucocorticoid receptor (PDB: 4P6X) revealed that the conformation was consistent with previous X-ray-diffraction studies [51] and revealed associated critical molecular interactions. Notably, HCT formed hydrogen bonds with Thr^739^, Asn^564^, and Arg^611^. Additionally, a hydrogen-bonded network is formed between water, Met^604^, and Arg^611^, providing for effective hydrogen-bonding interaction of Arg^611^ with HCT (Figure 9A). The importance of water molecules in ligand binding and affinity prediction has been characterized before [77,78]. On the mineralocorticoid target (PDB: 5HCV), HCT interacted through hydrogen bonding with the corresponding amino-acid residues that included Thr^945^ and Asn^770^. A similar water-based hydrogen-bonded network was observed encompassing Arg^817^ and Ser^810^. The similar binding profile of HCT on the glucocorticoid and mineralocorticoid receptors reinforces the literature reports that the ligand-binding domains of these two steroid receptors are homologous. However, these have distinct features that result into different ligand selectivities. While HCT binds on both glucocorticoid and mineralocorticoid receptors, the glucocorticoid receptor is more selective for this ligand [79]. In agreement with this, the results from this study produced a higher docking score of −13.026 kcal/mol for HCT glucocorticoid-receptor binding and −11.084 kcal/mol for the mineralocorticoid-receptor-binding potential.

**Figure 10:**
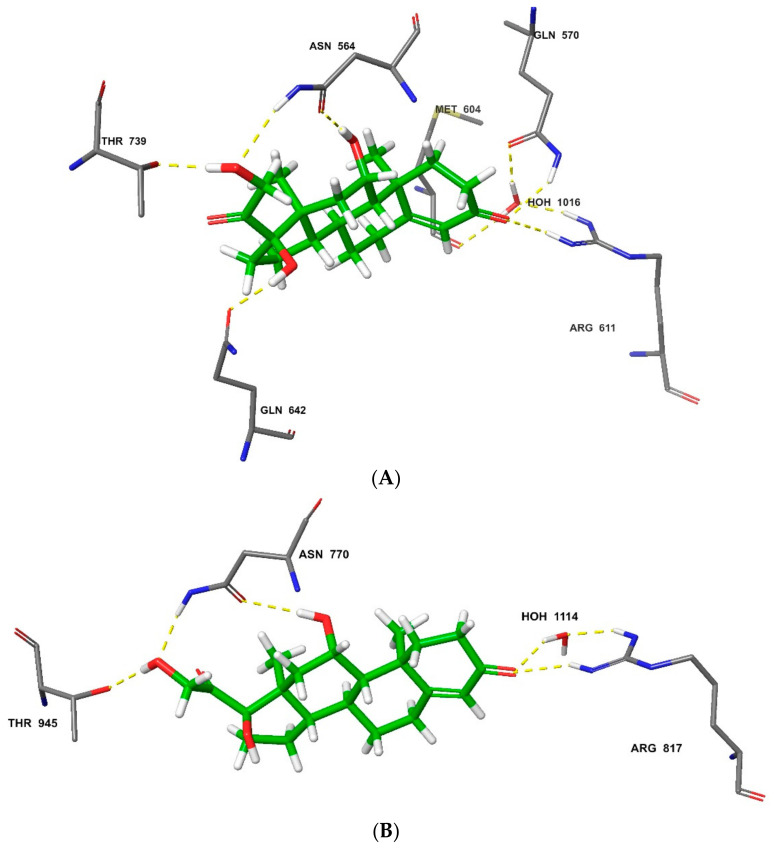
Molecular interactions in MM-GBSA re-docking of HCT against the glucocorticoid receptor with (**A**) MM-GBSA binding score: −79.38 kcal/mol; and mineralocorticoid receptor with (**B**) MM-GBSA binding score: −66.07 kcal/mol.

The MM-GBSA simulation revealed more intermolecular interactions between HCT and the receptors, with increased accuracy in binding-affinity prediction. This method showed a better differentiation of HCT binding potential between the two steroid receptors, whereby higher affinity (MM-GBSA-binding score: −79.38 kcal/mol) was obtained for the glucocorticoid receptor compared to the mineralocorticoid target (MM-GBSA-binding score: −66.07 kcal/mol). This proved that the MM-GBSA re-docking method is ideal for ligand-binding affinity and pose prediction [80], and that the glucocorticoid receptor is more selective to HCT than the corresponding mineralocorticoid target [9]. In addition to the hydrogen-bonding amino-acid residues identified in the initial extra-precision docking, MM-GBSA spotted extra others in glucocorticoid-receptor binding, which included Gln^642^ as well as Gln^570^, that also participate in the water-induced hydrogen-bonding network (Figure 10A). While no additional amino-acid residue was suggested by MM-GBSA in the mineralocorticoid receptor’s HCT docking, the water-molecule orientation was optimized to initiate formation of the hydrogen bond between the Arg^817^ residue and the ligand (Figure 10A).

### 3.9. Analysis of the Surface Morphology of the HCT-Loaded Nanoparticles Using SEM

**Figure 11:**
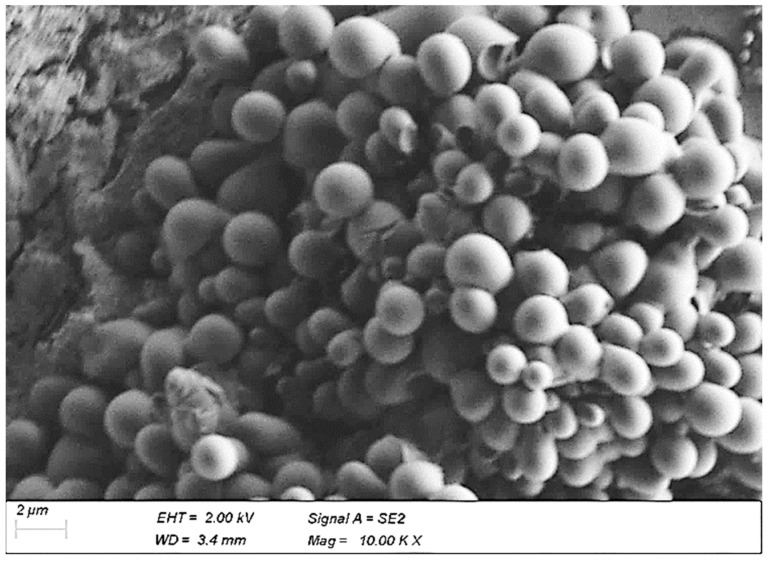
SEM micrograph prof the HCT-loaded SMS-PCL nanoparticles, confirming their polymeric structure.

The results of the SEM analysis demonstrated spherical shapes of the nanoparticles. This confirms that the HCT-loaded nanoparticles were synthesized and have polymeric structure, as shown in Figure 11’s SEM micrograph of the HCT-loaded SMS-PCL nanoparticles, confirming their polymeric structure. [77]. This further validated that the hydrophobic drug, HCT, was successfully incorporated within the nanoparticle structure. Additionally, the spherical structure shows self-aggregation of the polymer molecules—i.e., the hydrophilic “heads” of the polymer molecules face outside, while the hydrophobic “tails” are shielded inside the core of the micelle—thus shielding the contents of the micelle from the aqueous environment of the hydrogel [80].

Furthermore, this nanoparticle structure offers a considerable advantage in terms of topical delivery. Novel nanocarriers such as microemulsions, nanoemulsions, liposomes, and nanoparticulate carriers have been extensively studied for dermal and transdermal drug administration. As topical drug-delivery systems, nanoparticles offer several benefits, including increased drug deposition in the target region, improved physicochemical stability of the drug-loaded nanoparticles, and sustained and regulated drug administration from nanoparticulate systems [81]. This firmly places the synthesized SMS-PCL nanoparticles as excellent drug-delivery vehicles for the topical delivery of HCT in the treatment of PV.

### 3.10. In Vitro Cytotoxicity

**Figure 12:**
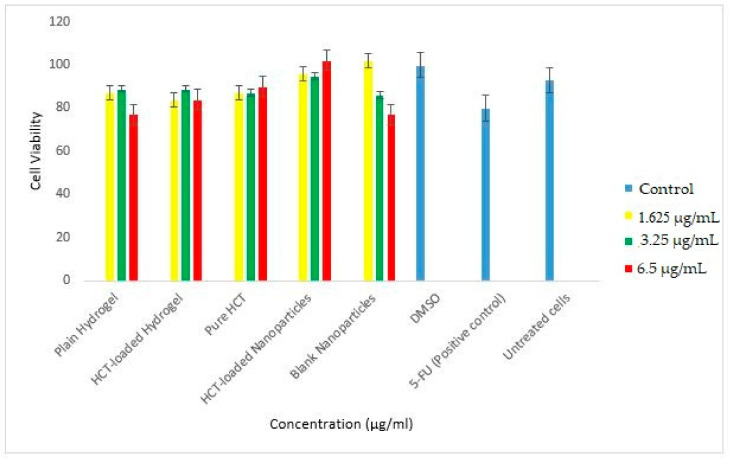
HaCaT cell viability (%) following treatment after 24 h. The graph shows cell viability following MTT assay of the treated cells against the controls.

**Figure 13:**
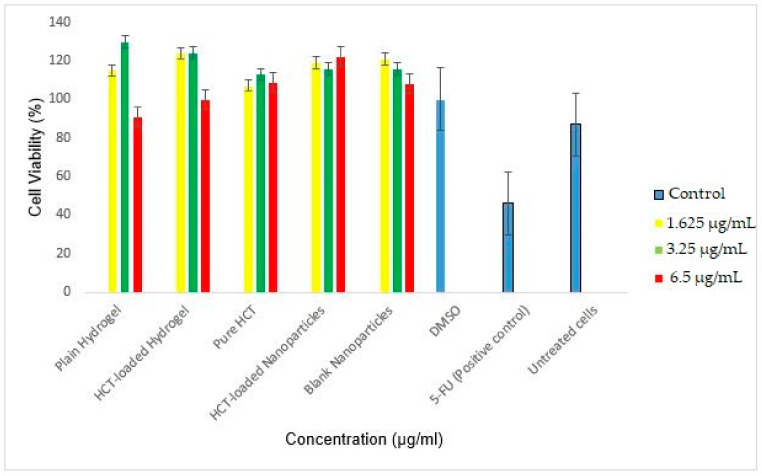
HaCaT cell viability (%) following treatment after 48 h. The graph shows cell viability following MTT assay of the treated cells against the controls.

In order to determine the safety and applicability of the thermos-responsive HCT-loaded hydrogel, cytotoxicity evaluations were conducted. In this study, the MTT assay data of the plain hydrogel, HCT-loaded hydrogel, pure HCT, HCT-loaded nanoparticles, and blank nanoparticles was collected at 24 and 48 h and depicted in Figure 12 and Figure 13, respectively. The viability of the treated cells at both time points was maintained. At 24 h (3.25 µg/mL), the viability of the HCT nanoparticles was 95%, whereas the HCT-loaded hydrogel showed viability of 89%, when compared against the positive control (5-FU) at 80%. After 48 h, the HCT-loaded nanoparticles and HCT-loaded hydrogel had high viability (116% and 124%, respectively, at 3.25 µg/mL) vs. 5-FU (46%). The loaded hydrogel demonstrated a slight decrease in cell viability when compared to the HCT nanoparticles alone.

A dose–response relationship is observed with respect to the plain hydrogel and blank nanoparticles—the higher the concentration is, the lower the cell viability. However, this is reversed for the HCT-loaded nanoparticles, where the concentration of the drug is directly proportional to the cell viability. The ability for the treated cells to maintain viability alludes to the biocompatibility of the polymers used in the synthesis of the nanoparticles as well as the hydrogel carrier. Furthermore, it demonstrates the applicability of the thermos-responsive hydrogel in the treatment of inflammatory skin conditions.

**Figure 14:**
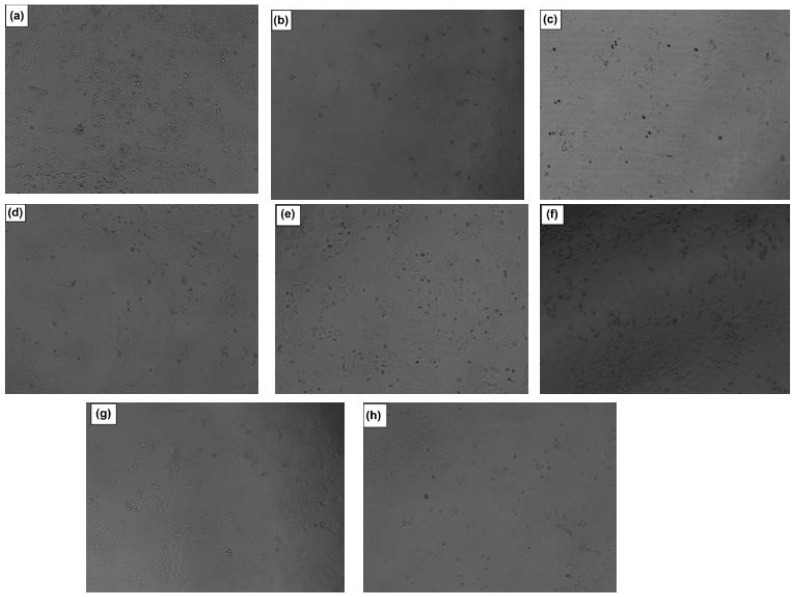
Light-microscopy images of the (**a**) plain hydrogel, (**b**) HCT-loaded hydrogel, (**c**) pure HCT, (**d**) HCT-loaded nanoparticles, (**e**) blank nanoparticles, (**f**) 5-FU, (**g**) untreated cells, and (**h**) DMSO control after 48 h treatment. The scale bar is 100 µm.

Microscopic analysis demonstrated the internalization of the HCT-loaded nanoparticles, blank nanoparticles, pure HCT, HCT-loaded hydrogel, plain hydrogel, and the controls (5-FU, DMSO, and untreated cells) (Figure 14).

## 4. Conclusions

PV is a chronic, incurable, inflammatory skin condition with a severe impact on quality of life. In this present study, a thermos-responsive polymeric-hydrogel system loaded with an anti-psoriasis drug, HCT, was synthesized. Nanoparticles with a diameter of 110.5 nm, zeta potential −58.7 mV, and PdI of 0.15 were successfully synthesized and loaded with HCT (DEE = 76%). SEM analysis confirmed the spherical shape of the NLCs. UV/Vis spectroscopy (λ = 245 nm) confirmed the linear correlation between the concentration of the drug and absorbance. Physicochemical characterizations of the drug-loaded and non-drug-loaded NLCs, as well as of the pure HCT drug, were performed using DSC, FTIR, and TGA—these confirmed the presence of HCT within the nanoparticle structures. A thermos-responsive hydrogel with SGTT ~35 °C was successfully synthesized. Drug-release profiles for HCT were controlled by the pH of the release medium. The release of HCT was slower and more sustained from the CMC thermo-responsive hydrogel in comparison to the free nanoparticles. HCT interacts with steroid receptors in the skin to reverse the inflammation and proliferation associated with PV. Our in silico study confirmed the high-affinity potential of HCT for the glucocorticoid and mineralocorticoid receptors. In vitro cytotoxicity results showed that the synthesized HCT-loaded, thermo-responsive hydrogel system is safe and tolerable for application to the skin, due to the high cell viability demonstrated by both the HCT-loaded nanoparticles as well as the HCT-loaded hydrogel, which was 116% and 124%, respectively. Microscopic studies show an internalization of the hydrogel system, showing the biocompatibility and sustained release of the system. Further studies with in vivo models should be conducted to assess this as a potential novel therapeutic outlet for the treatment of inflammatory skin disease.

## Figures and Tables

**Table 1:** HCT drug-release profiles at pH 5.2.

HCT-Loaded Nanoparticles				
Time (h)	Absorbance (nm)	Concentration (µg/mL)	Mass (mg)	% Release
0	0.042 ± 0.004	0.837	0.046	0.230
0.25	0.065 ± 0.004	1.067	0.059	0.293
0.5	0.071 ± 0.004	1.123	0.062	0.309
1	0.085 ± 0.003	1.262	0.069	0.347
2	0.101 ± 0.003	1.420	0.078	0.390
4	0.186 ± 0.004	2.263	0.124	0.622
6	0.215 ± 0.007	2.546	0.140	0.700
24	0.250 ± 0.009	2.899	0.159	0.797
**HCT-Loaded CMC Hydrogel**				
**Time (h)**	**Absorbance (nm)**	**Concentration** **(µg/mL)**	**Mass (mg)**	**% Release**
0	0.013 ± 0.002	0.557	0.031	0.153
0.25	0.056 ± 0.002	0.975	0.054	0.268
0.5	0.062 ± 0.002	1.038	0.057	0.285
1	0.081 ± 0.001	1.222	0.067	0.336
2	0.093 ± 0.004	1.347	0.074	0.370
4	0.172 ± 0.003	2.128	0.117	0.585
6	0.190 ± 0.002	2.299	0.126	0.632
24	0.242 ± 0.002	2.813	0.155	0.774

**Table 2:** HCT drug-release profiles at pH 5.9.

HCT-Loaded Nanoparticles				
Time (h)	Absorbance (nm)	Concentration (µg/mL)	Mass (mg)	% Release
0	0.053 ± 0.002	0.945	0.052	0.260
0.25	0.079 ± 0.007	1.202	0.066	0.331
0.5	0.102 ± 0.004	1.433	0.079	0.394
1	0.132 ± 0.002	1.729	0.095	0.476
2	0.185 ± 0.005	1.256	0.124	0.620
4	0.203 ± 0.003	2.428	0.134	0.668
6	0.229 ± 0.005	2.691	0.148	0.740
24	0.286 ± 0.005	3.248	0.179	0.839
**HCT-Loaded CMC Hydrogel**				
**Time (h)**	**Absorbance (nm)**	**Concentration (µg/mL)**	**Mass (mg)**	**% Release**
0	0.053 ± 0.002	0.945	0.047	0.236
0.25	0.079 ± 0.007	1.202	0.063	0.313
0.5	0.102 ± 0.004	1.433	0.071	0.356
1	0.132 ± 0.002	1.729	0.093	0.465
2	0.185 ± 0.005	1.256	0.099	0.494
4	0.203 ± 0.003	2.428	0.127	0.633
6	0.229 ± 0.005	2.691	0.141	0.707
24	0.286 ± 0.005	3.248	0.164	0.819

## Data Availability

Not applicable.
